# Value of Post-/Pre-Procedural Aortic Regurgitation Ratio vs. Pre-Procedural Aortic Valve Calcium Score to Predict Moderate to Severe Paravalvular Leak Requiring Post-Dilation after Transcatheter Aortic Valve Implantation

**DOI:** 10.3390/jcm12247735

**Published:** 2023-12-17

**Authors:** Roman Uebelacker, Simon S. Martin, Mariuca Vasa-Nicotera, Silvia Mas-Peiro

**Affiliations:** 1 Department of Cardiology, University Hospital Frankfurt am Main, Theodor-Stern-Kai 7, 60590 Frankfurt am Main, Germany; romanuebelacker@web.de (R.U.); mariuca4101@gmail.com (M.V.-N.); 2 Department of Radiology, University Hospital Frankfurt am Main, 60590 Frankfurt am Main, Germany; simon.martin@kgu.de; 3 German Centre for Cardiovascular Research (DZHK), 10785 Berlin, Germany; 4 Cardiopulmonary Institute (CPI), 60590 Frankfurt am Main, Germany

**Keywords:** aortic valve stenosis, transcatheter aortic valve implantation, aortic regurgitation index, calcification, post-dilation

## Abstract

Background and aim: Tools that assist interventionists in selecting patients for post-dilation (PD) are needed. We aimed to assess whether pre-interventional aortic valve calcium (AVC) or the peri-interventional aortic regurgitation (ARI) ratio is a better predictor for a more than mild paravalvular leak (PVL) requiring PD after TAVI. Methods: Patients undergoing TAVI with available data on AVC derived from MSCTs and the ARI ratio derived from peri-interventional hemodynamic curves were studied. The main outcome was moderate-to-severe PVL requiring PD. Results: In 237 patients, more than mild PVL after valve deployment was present in 25.7%. PD was performed in 65 patients. The median (IQR) total AVC was 390.5 (211.5–665.4) mm^3^. All calcification values were significantly higher in patients who underwent PD. The median (IQR) individual threshold was 600 (550–685) Hus. The overall ARI ratio was 0.78 (0.61–0.96), with values being significantly lower in patients who underwent PD: 0.61 (0.49–0.80) vs. 0.82 (0.69–0.99) (*p* < 0.001). Both the ARI ratio (OR [95%CI] 0.053 [0.014–0.203]; *p* < 0.001) and AVC (1.01 [1.000–1.002]; *p* = 0.015) predicted PD need. ROC curves showed higher discrimination for the ARI ratio (AUC 0.73) than for any calcification parameter (all AUCs ≤ 0.62). Conclusions: The ARI ratio provides interventionists with a powerful predictive tool for PVL requiring PD after TAVI that is beyond the predictive value of pre-procedural valve calcification derived from MSCT.

## 1. Introduction

More than mild paravalvular leak (PVL) has been shown to be associated with poor short- and long-term outcomes in patients with aortic stenosis undergoing transcatheter aortic valve implantation (TAVI) [1,2,3]. Significant PVL often requires post-dilation (PD), which allows for a reduction in the PVL degree by obtaining a full expansion of the prosthesis [4], provided that the implantation depth is appropriate. Previous studies have shown that PD is performed in a significant percentage of TAVI procedures, both with self-expandable and balloon-expandable valves (10.4–40.0%) [5]. However, PD has been associated with severe complications, particularly with a high incidence of stroke [5,6]; therefore, a careful assessment is needed when selecting patients who should undergo such a corrective measure.

Several factors have been shown to predict the need for PD to minimize PVL. The degree of valve calcification measured with multi-slice computer tomography (MSCT) has been considered one the most important parameters predicting the need for PD [4,7,8]. The burden of calcium can be used to identify patients with a poor prognosis, and this may lead to a surgical approach in severe cases. Other authors have suggested an added value of intraprocedural hemodynamic measures as a guide for post-dilation during TAVI procedures, with an impact on mortality [9]. There is an unmet need for tools to assist operators with such decision-making.

Thus, the aim of this study was to assess whether the pre-interventional aortic valve calcium (AVC) score or the peri-interventional aortic regurgitation ratio was a better predictor for a more than mild PVL requiring PD. 

## 2. Methods

### 2.1. Study Population

Patients who underwent TAVI from March 2016 to March 2019 with available data on AVC derived from MSCTs and an aortic regurgitation index (ARI) ratio estimation based on peri-interventional hemodynamic curves were included in this study. Patients with low-quality MSCTs, missing hemodynamic values, or a prior biological valve preventing accurate calcification assessment were excluded from the analysis.

### 2.2. Patient Characteristics, Procedural Parameters, and Post-Procedural Outcomes

Demographic and clinical parameters were collected at baseline for all patients. Follow-up was performed with outpatient visits or telephone interviews with patients or family members. All procedures were performed after assessment by the local heart team. Both self-expandable (Portico, Evolut R, Symetis Accurate) and balloon-expandable (Sapien 3) devices were implanted. Most patients underwent TAVI under local anaesthesia and under rapid pacing. The severity of PVL was assessed peri-procedurally using angiography or echocardiography, and the decision for PD was made individually by each operator based on clinical judgement. 

### 2.3. Calcification Measurements Using MSCT

**Figure 1:**
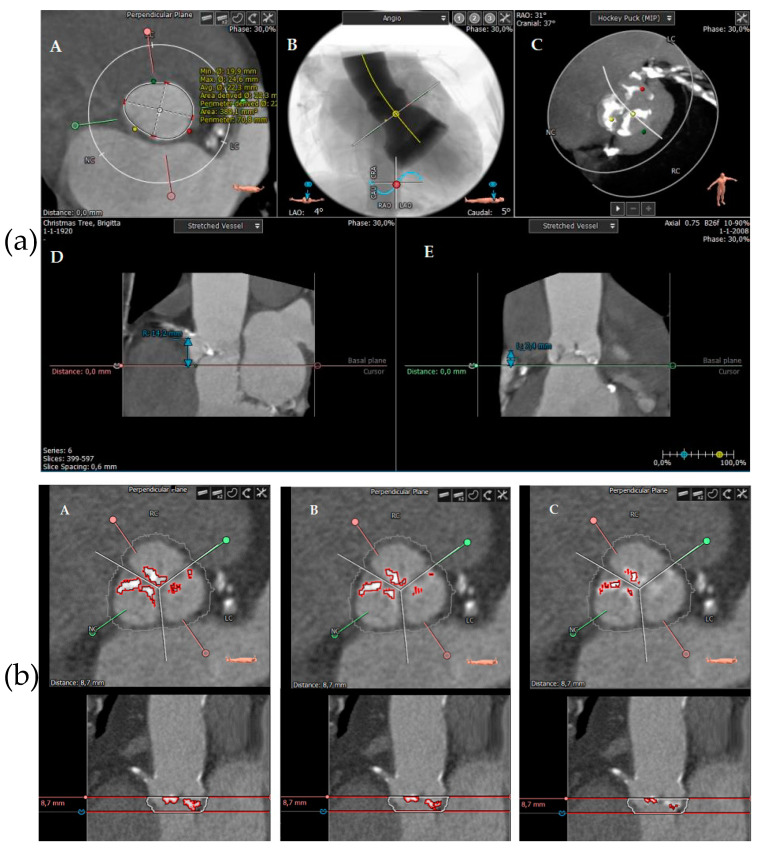
(**a**) MSCT measurements using the 3mensio program. From left to right: (**A**) measurement of the annulus, (**B**) angulation, (**C**) hockey puck showing a moderate degree of calcification, (**D**) height of right coronary artery, and (**E**) height of left coronary artery. (**b**) MSCT measurements using different HU thresholds. From left to right: (**A**) 500 HUs, (**B**) 600 HUs (individualized), and (**C**) 800 HUs.

All patients underwent pre-procedural MSCT (Somatom Force, Siemens Healthineers, Forchheim, Germany, with collimation: 2 mm × 192 mm × 0.6 mm) for TAVI planning using a prospective high-pitch spiral electrocardiographic (ECG) gating technique. This included prosthesis sizing as well as quantification and distribution of valve calcification. Contrast enhancement was injected according to local protocol using 70 mL of contrast agent (Iomeprol 400 mg/mL, Bracco, Milan, Italy) followed by saline 40 mL at a 5 mL/s injection rate. A dedicated software tool (3mensio Structural Heart software 10.0, Pie Medical Imaging, Maastricht, The Netherlands) was used for calcification measures [10]. Images were reconstructed using multiplanar reformations. Analyses were performed by experienced technicians trained in MSCT assessment, who were blinded to the procedural data. A virtual ring with three anchor points at the bases of the aortic leaflets was used to characterize the aortic annulus in an axial plane [11]. In addition to the anatomical baseline measurements (i.e., annulus perimeter), total AVC was quantified. The region of interest was defined from the aortic annulus plane to the leaflet tips based on prior publications [7,8,12,13]. Calcification was also measured per cusp: RCC (right coronary cusp), LCC (left coronary cusp), and NCC (non-coronary cusp). Left ventricular outflow tract (LVOT) calcification was measured separately. Since a threshold to detect calcium on contrast-enhanced scans has not yet been standardised in the literature, the calcification score was based on a dynamic threshold based on luminal attenuation measured in Hounsfield units (Hus), as previously proposed by Bettinger et al. [12]. This patient-specific approach allows for better calcium discrimination compared with various fixed-intensity thresholds (e.g., 500 Hus or 850 Hus). The eraser function was used to manually eliminate any contrast detected as calcification. An example of such measurements is shown in Figure 1a,b.

### 2.4. Hemodynamic Measurements

Hemodynamic curves were registered before and after every procedure both in the left ventricle and in the ascending aorta. As previously described by Sinning et al., since diastolic function may be affected by rapid pacing, which is required during the procedure, all post-procedural measurements were performed some minutes after deployment of the valve. A systematic recording of pressure curves was applied using a minimum of three cardiac cycles with normal heart rate [14]. 

**Figure 2:**
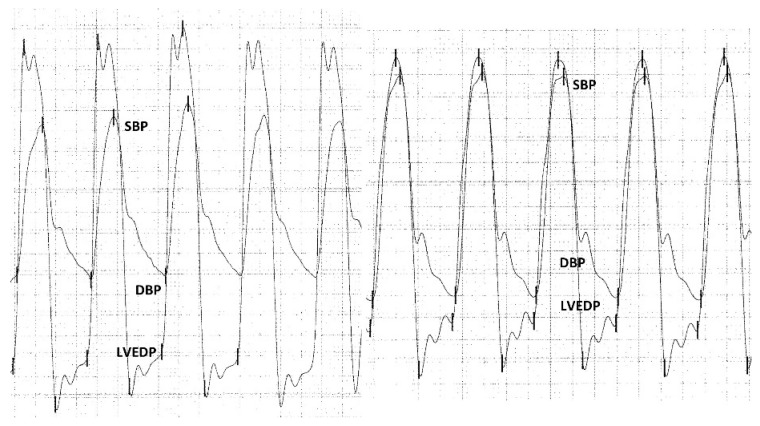
Hemodynamic measurements and calculation of the aortic regurgitation index ratio. Calculation of the AR index before TAVI: ([DBP − LVEDP]/SBP) × 100 = ([58 − 19]/138) × 100 = 28. The AR index before TAVI is 26. Calculation of the AR index post-initial TAVI: ([DBP − LVEDP]/SBP) × 100 = ([50 − 35]/148) × 100 = 10. The AR index post-initial TAVI is 12. Calculation of the post-initial TAVI/pre-procedural ARI ratio: AR index post TAVI/AR index pre TAVI = 10/28 = 0.36. AR: aortic regurgitation; DBP: diastolic blood pressure; LVEDP: left ventricular end-diastolic pressure; SBP: systolic blood pressure.

Pressure curve analyses were performed retrospectively. ARI was calculated as follows: ([diastolic blood pressure − left ventricular end-diastolic pressure]/systolic blood pressure) × 100. The ARI ratio was calculated as follows: post-procedural ARI/pre-procedural ARI. An example of such calculations is shown in Figure 2. 

The Ethics Committee of the University Hospital of Frankfurt approved the study protocol (296/16), and written informed consent was obtained from all patients.

### 2.5. Study Endpoints

The main study endpoint was moderate-to-severe PVL requiring PD after TAVI. Secondary outcomes included complications according to VARC 2 criteria, 30-day mortality, and 1-year mortality. 

Additionally, taking into account that pre-procedural valve dilation may be helpful to prepare the ground for valve implantation, we also assessed the residual regurgitation in the subgroup of patients with high calcification (i.e., severe visual degree of calcification) who underwent pre-dilation and compared their results with those in patients with severe calcification not undergoing pre-dilation.

### 2.6. Statistical Analysis

The mean ± standard deviation or the median (IQR) was reported for continuous variables, based on their normal or non-normal distribution. Frequencies were used to report categorical variables. The Mann–Whitney U test was used to compare independent non-parametric variables, whereas the Student’s *t*-test was used for parametric variables. Frequencies were compared using the chi-squared test. Binary logistic regression was performed to assess the impact of hemodynamic and calcification parameters on PD. Several receiver operating characteristic curves (ROC) were created to determine the area under the curve (AUC) for both hemodynamic and calcification parameters. Statistical significance was set at *p* < 0.05. All analyses were performed with the SPSS statistical software package (IBM SPSS, version 24.0, IBM Corporation, Armonk, NY, USA) and the software program BiAS (BiAS version 11.12, Epsilon Verlag, Frankfurt, Germany).

## 3. Results

### 3.1. Study Population 

**Figure 3:**
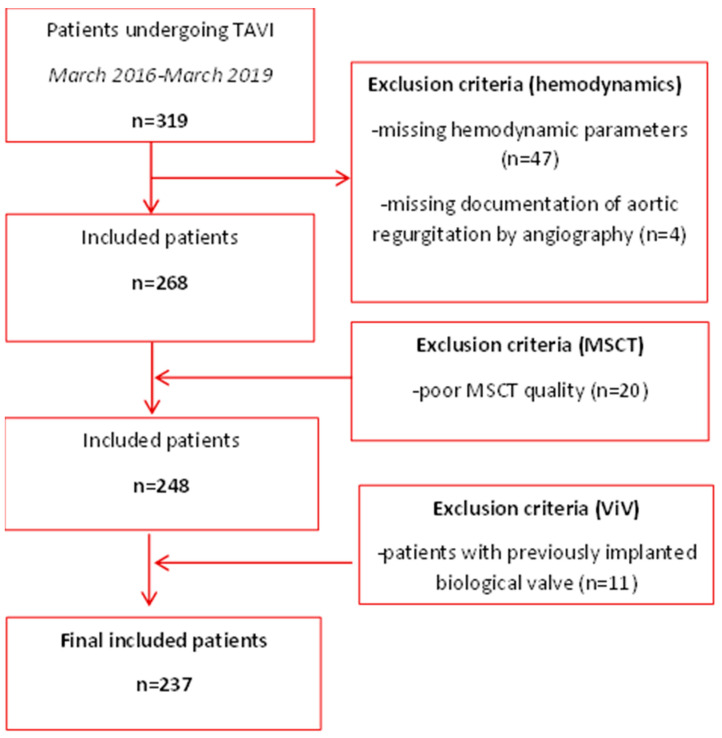
Flowchart of the study population.

A total of 237 patients with complete MSCT, angiographic, and hemodynamic data were included in this study. A flow chart of the study population is shown in Figure 3. 

### 3.2. Patient Characteristics, Procedural Parameters, and Post-Procedural Outcomes

The overall median (IQR) age was 82 (78–85) years, and 57% of the patients were male. The most frequent comorbidities were hypertension (88.61%), atrial fibrillation (45.57%), and previous percutaneous coronary intervention (PCI) (42.19%). The baseline New York Heart Association (NYHA) class was III/IV in 80.9%. 

The majority of patients underwent initial pre-dilation (74.3%). Implanted devices were the following: 35.4% Symetis Acurate Neo (Boston Scientific, Marlborough, MA, USA), 30.4% Portico (Abbott Vascular, Abbott Park, IL, USA), 1.4% Evolut R (Medtronic), and 22.8% Sapien 3 (Edwards Lifesciences, Irvine, CA, USA). Angiographically measured more than mild PVL directly after valve deployment was present in 25.7% of patients. Post-dilation was performed on a total of 65 patients. One patient required snearing of the device due to too deep positioning of the prosthesis into the LVOT. No patients required the implantation of a second prosthesis (valve in valve). More than mild PVL after corrective measures was 7.6%, as measured with post-procedural echocardiography.

The most frequent overall post-procedural complications were the need for a permanent pacemaker (13.9%), followed by major vascular complications (5.1%) and life-threatening or major bleeding (5.1%). The overall 30-day and 1-year mortalities were 3.0% and 17.7%, respectively. 

There were no differences in the baseline clinical characteristics when comparing patients who underwent PD to those who did not require such a corrective measure. Regarding procedural results, PD was more often performed when using Symetis Acurate Neo and Evolut R prostheses. As expected, contrast dye use, fluoroscopy time, and procedure length were significantly higher in patients who underwent PD. Minor stroke and cardiac tamponade were also more common in the latter group. In-hospital, 30-day, and 1-year mortality did not differ between groups. The data are shown in Table 1 and Table 2.

### 3.3. Calcification Measurements Using MSCT

The overall median (IQR) perimeter-derived annulus was 25.5 (23.5–27.6) mm. The visually assessed AVC results for all patients showed the following distribution: none (1.7%), mild (19.4%), moderate (41.8%), and severe (37.1%). The median (IQR) total AVC score was 390.5 (211.5–665.4) mm^3^. The median AVC per leaflet was: 107.3 (49.8–209.6) mm^3^ for LCC, 89.9 (50–191.8) mm^3^ for RCC, and 154.1 (83.3–296.6) mm^3^ for NCC. The median (IQR) for LVOT calcification was 5.2 (0.1–42) mm^3^. All calcification values were significantly higher in patients who underwent a PD compared with those who did not. The median (IQR) individual threshold was 600 (550–685) HUs. Further details are shown in Table 3.

### 3.4. Hemodynamic Measurements

The overall median (IQR) ARI ratio was 0.78 (0.61–0.96). As expected, the ARI ratio values were significantly lower in patients who underwent PD: 0.61 (0.49–0.80) vs. 0.82 (0.69–0.99) (*p* < 0.001). When using the previously established ARI ratio threshold [14,15], a significantly higher frequency of patients who underwent corrective interventions showed an ARI ratio < 0.6 (49.2% vs. 13.4%, *p* < 0.001). Further detailed hemodynamic measurements are shown in Table 4. 

### 3.5. Impact of Calcification and Hemodynamic Measurements on PD

The ARI ratio decreased with increasing AVC categories: 1.11 (0.81–2.01) for no calcification, 0.80 (0.61–0.93) for mild calcification, 0.79 (0.62–0.97) for moderate calcification, and 0.74 (0.57–0.93) for severe calcification. Using the ARI threshold of 0.6, the AVC score was higher in patients below such threshold: 407.8 (273.4–611.80) mm^3^ vs. 374.7 (207.58–670.78) mm^3^. However, this difference did not achieve significance (*p* = ns).

**Figure 4:**
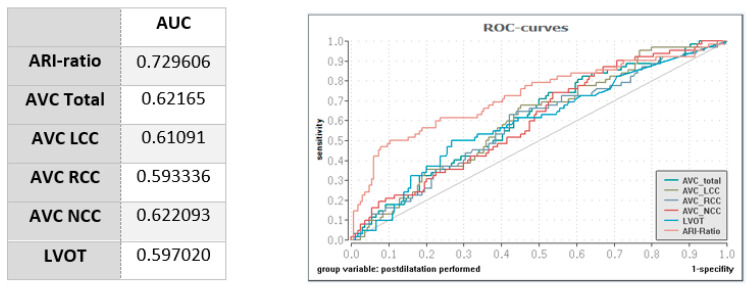
Receiver operating curves for hemodynamic measures and calcification parameters. ARI: aortic regurgitation index; AUC: area under the curve; AVC: aortic valve calcification; LVOT: left ventricular outflow tract.

Binary logistic regressions showed a lower ARI ratio and a higher total AVC score to significantly predict the need for PD due to PVL: OR 0.053, 95% CI 0.014–0.203; *p* < 0.001, and OR 1.01, 95% CI 1.000–1.002; *p* = 0.015, respectively. However, the ROC curves showed a higher discrimination for the ARI ratio (AUC 0.73) than for all tested calcification parameters (all AUCs ≤ 0.62) (see Figure 4). 

### 3.6. Residual PVL in Patients with High Calcification Undergoing Pre-Dilation

The visual degree of calcification was severe in 88 out of 237 patients (37.1%) (Table 3). Sixty-nine out of these eighty-eight patients (78.4%) underwent pre-dilation. PD was required as a corrective measure for PVL in 27/69 pre-dilated patients (39.1%) and 42/69 pre-dilated patients (60.9%) who did not receive PD (n.s.).

## 4. Discussion

Our data demonstrate that: (1) an elevated AVC score derived from MSCT before TAVI predicts the need for more than mild PVL requiring PD; (2) peri-interventional hemodynamic parameters, and particularly, a low ARI ratio, is also a powerful predictive tool for the need for PD after valve deployment; (3) the ARI ratio is a better discriminator for corrective measures than baseline calcification; and (4) pre-dilation in patients with a severe burden of calcium did not seem to have a significant impact on residual PVL, as assessed by the need for corrective PD.

In daily practice, since PD is mostly performed at the operator’s discretion, there is a lack of consensus on when to perform corrective measures for PVL after valve deployment, with regurgitation itself making the interventionist use PD. A higher frequency in severe complications such as stroke or cardiac tamponade due to annulus rupture, as well as a higher need for contrast dye and a longer procedure time are usually associated with PD, as confirmed by our study. Therefore, a strict selection of PD candidates is crucial.

Although measurements of calcification using MSCT prior to the procedure may provide some hints on whether PD will be needed, an intra-procedural confirmation may be reassuring. It is a well-known fact that the evaluation of prosthetic valve regurgitation presents technical difficulties [16]. Both echocardiography and angiography are usually used during the procedure to assess PVL immediately after deployment of the valve. However, due to the subjective nature of both modalities, a more observer-independent tool such as hemodynamic assessment may be helpful.

Each of the two modalities assessed in the present study shows its own unique advantages. 

MSCT provides not only a quantitative but also a qualitative evaluation, including anatomic distribution of the calcification [17]. Additionally, it enables the operator to anticipate the need for PD a priori, depending on the amount and localization of calcification. The current main challenge when using MSCT is the lack of a standardized threshold for calcium detection. While calcification can be overlooked in images with extremely low attenuation, it may be overestimated in images with high attenuation, causing contrasted regions to be misleadingly classified as calcium. The interaction of several factors can influence the degree of opacification including radiation dose, contrast concentration and volume, or a patient’s body composition [18]. For this reason, different thresholds have been used in the past. While some authors have shown a lower empiric threshold to predict mortality (i.e., 350 HU) [19], Seiffert et al. used a threshold of 500 HUs [20], and Jilaihawi et al. and Fonseca et al. demonstrated a threshold of 850 HUs to be the best predictor for PVL [13,21]. In the present study, we used an “individualized” threshold based on an analysis performed by Bettinger and colleagues suggesting the use of a dynamic threshold based on luminal attenuation instead of fixed-intensity values, as this was shown to be a better predictor for PVL [12]. In fact, a future threshold standardization to detect calcium will certainly be beneficial in daily clinical practice. 

As for hemodynamic assessment, the ARI ratio offers an accurate quantification immediately after valve deployment, providing real-time information for decisions on PD. Such catheter-based hemodynamic indices may present an additional benefit in patients in whom PVL is technically difficult to assess either echocardiographically or angiographically. In fact, with newer generation valves aiming at reducing PVL post-TAVI, a quantitative tool such as the ARI ratio may enable the detection of even milder PVL [9]. Lastly, hemodynamic measurements do not require the use of contrast dye, which can be a significant advantage in patients presenting with prior renal failure. Its limitations include inaccuracy of the measurements due to the heart rate or the presence of a concomitant diastolic dysfunction.

Several previous studies have assessed the impact of hemodynamic indices on prognosis after TAVI. With the majority of studies showing a significant association with survival [14,22,23,24,25], two investigations from the same centre recently suggested contradictory findings regarding short- and long-term outcomes on mortality [26,27]. However, to our knowledge, only one study showed the impact of a time-integrated aortic regurgitation index (TIARI) on PVL requiring PD [9]. Our study confirms the predictive value of hemodynamic parameters (i.e., the ARI ratio) on PD, suggesting that although such values may not always predict mortality after TAVI, they may be extremely useful to guide PD immediately after valve deployment. A real-time quantitative measure may help operators discriminate whether the benefit of a PD outweighs the potential risk of associated severe complications (i.e., annulus rupture or stroke) [28].

Although several previous studies have assessed the correlation between different peri-interventional modalities of PVL evaluation (echocardiography, angiography) and hemodynamic parameters, to this day, none has assessed the association between MSCT-derived calcification degree and hemodynamic indices regarding the need for PD. A previous study by Kumar et al. showed an added incremental value of PVL assessment with the time-integrated aortic regurgitation index (TIARI) over angiographic and echocardiographic evaluation when deciding to perform PD [9]. 

Since the ARI ratio was shown to be a better predictor for PD and due to the fact that hemodynamic calculations have not yet reached widespread use in clinical practice, we suggest including this parameter in the decision-making process for PD. A multimodal approach combining both imaging modalities (calcification derived from MSCT and visually assessed PVL in echocardiography and angiography) and peri-interventional hemodynamic measurements should be able to provide operators with enough qualitative and quantitative information for guidance on corrective measures. 

### Limitations

Several limitations need to be acknowledged. First, this is a single-centre study; thus, extrapolation to other populations should be performed cautiously. Second, MSCT measurements were performed using a visually assessed calcification score. Although a HU threshold has not been formally established yet, further analyses will need to be performed once a strict fixed-intensity threshold (i.e., 500 HUs, 850 HUs) has been identified. Third, since hemodynamic curves were recorded during clinical routine, a selection bias towards more stable patients in the catheterization lab cannot be excluded. Patients with acute complications may have been left out of hemodynamic measurements in emergency situations. Fourth, the majority of implanted prostheses in our study were self-expanding valves. Further studies with a higher number of balloon-expandable and possibly new-generation valves are needed. Fifth, the use of ARI may be inaccurate in patients with a rather low pre-procedural regurgitation and a very stiff calcified annulus. In such patients, PD may be scarcely useful in spite of a rather high ARI value. Lastly, although we used PD as an endpoint due to its objective nature, PVL measured angiographically or using echocardiography could have been an alternative despite its intra- and interobserver variability.

## 5. Conclusions

The ARI ratio appears to provide interventionists with a powerful predictive tool for PVL requiring PD after TAVI, which is beyond the predictive value of pre-procedural valve calcification derived from MSCT. A multimodal approach including quantitative and qualitative approaches may be helpful for guidance on PD after valve deployment. 

## Figures and Tables

**Table 1:** Baseline characteristics overall and in patients with and without post-dilation.

	All Patients (*n* = 237)	Without Post-Dilation (*n* = 172)	With Post-Dilation (*n* = 65)	*p*-Value
Age (years)	82 (78–85)	81.5 (78–85)	83 (79.5–85)	0.17
Female (%)	102 (43%)	75 (43.6%)	27 (41.5%)	0.774
BMI (kg/m^2^)	26.8 (24.3–30.4)	26.7 (24.5–30)	27.3 (23.4–30.9)	0.931
NYHA I II III IV	(*n* = 236) 0 (0%) 45 (19.1%) 169 (71.6%) 22 (9.3%)	(*n* = 171) 0 (0%) 28 (16.4%) 126 (73.7%) 17 (9.9%)	0 (0%) 17 (26.2%) 43 (66.2%) 5 (7.7%)	0.109
Euro Score II	3.13 (2.16–6.03)	3.53 (2.26–6.1)	2.77 (2.02–5.55)	0.109
STS Score	3.07 (2.26–4.68)	3.07 (2.13–5.34)	3.17 (2.41–4.24)	0.935
CAOD	39 (16.5%)	31 (18%)	8 (12.3%)	0.291
PAOD	33 (13.9%)	23 (13.4%)	10 (15.4%)	0.69
s/p heart surgery	21 (8.9%)	17 (9.9%)	4 (6.2%)	0.367
COPD	45 (19%)	34 (19.8%)	11 (16.9%)	0.618
Insulin dependent diabetes mellitus	69 (29.1%) 22 (9.3%)	51 (29.7%) 15 (8.7%)	18 (27.7%) 7 (10.8%)	0.767 0.628
Hypertension	210 (88.6%)	151 (87.8%)	59 (90.8%)	0.52
s/p myocardial infarction	42 (17.7%)	32 (18.6%)	10 (15.4%)	0.562
s/p stroke	29 (12.2%)	22 (12.8%)	7 (10.8%)	0.672
s/p TIA	8 (3.4%)	7 (4.1%)	1 (1.5%)	0.337
s/p percutaneous coronary intervention	100 (42.2%)	74 (43.0%)	26 (40.0%)	0.674
CAD none 1 Vessel CAD 2 Vessel CAD 3 Vessel CAD	80 (33.8%) 61 (25.7%) 40 (16.9%) 56 (23.6%)	55 (32%) 40 (23.3%) 32 (18.6%) 45 (26.2%)	25 (38.5%) 21 (32.3%) 8 (12.3%) 11 (16.9%)	0.079
s/p pacemaker	32 (13.5%)	24 (14%)	8 (12.3%)	0.741
Atrial fibrillation	108 (45.6%)	83 (48.3%)	25 (38.5%)	0.177
LVEF (%)	60 (50–60) (*n* = 234)	60 (50–60) (*n* = 170)	60 (50–65) (*n* = 64)	0.369

CAD: coronary artery disease; CAOD: cerebral arterial occlusive disease; COPD: chronic obstructive pulmonary disease; LVEF: left ventricular ejection fraction; PAOD: peripheral arterial occlusive disease; TIA: transient ischaemic attack.

**Table 2:** Procedural parameters overall and in patients with and without post-dilation.

	All Patients (*n* = 237)	Without Post-Dilation (*n* = 172)	With Post-Dilation (*n* = 65)	*p*-Value
Procedural parameters				
Valve type Portico™ Symetis Acurate™ Sapien 3™ Evolut R™	72 (30.4%) 84 (35.4%) 54 (22.8%) 27 (11.4%)	53 (30.8%) 48 (27.9%) 54 (31.4%) 17 (9.9%)	19 (29.2%) 36 (55.4%) 0 (0%) 10 (15.4%)	**<0.001**
Pre-dilation	176 (74.3%)	122 (70.9%)	54 (83.1%)	0.056
Contrast dye use (mL)	100 (70–140)	90 (70–130)	130 (100–155)	**<0.001**
Procedure duration (min)	60 (45–60) (*n* = 236)	45 (45–60) (*n* = 171)	60 (45–60)	**0.049**
Fluoroscopy time (min)	14.0 (10–18.8) (*n* = 235)	12.7 (9.45–18.5) (*n* = 171)	16.5 (13.9–19.6) (*n* = 64)	**<0.001**
Simultaneous coronary intervention	5 (2.1%) (*n* = 236)	4 (4.0%)	0 (0%)	0.168
Post-procedural complications				
Myocardial infarction	0 (0.0%)	0 (0.0%)	0 (0.0%)	ns
Stroke -Minor -Major	3 (1.3%) 7 (3.0%)	0 (0%) 5 (2.9%)	3 (4.6%) 2 (3.1%)	**0.005** 0.945
Bleeding (major or life-threatening)	12 (5.1%)	9 (5.2%)	3 (4.6%)	0.847
Pacemaker implantation	33 (13.9%)	26 (15.1%)	7 (10.8%)	0.388
Major vascular complication	12 (5.1%)	8 (4.7%)	4 (6.2%)	0.638
Cardiac tamponade	4 (1.7%)	1 (0.6%)	3 (4.6%)	**0.032**
Short- and mid-term survival				
In-hospital mortality	2 (0.8%)	1 (0.6%)	1 (2.1%)	0.473
30-day mortality	7 (3.0%)	6 (3.5%)	1 (1.5%)	0.429
One-year mortality	42 (17.7%)	26 (15.1%)	16 (24.6%)	0.088

**Table 3:** MSCT measurements overall and in patients with and without post-dilation.

	All Patients (*n* = 237)	Without Post-Dilation (*n* = 172)	With Post-Dilation (*n* = 65)	*p*-Value
Basic MSCT parameters				
Annulus Perimeter derived (mm)	25.49 ± 2.94	25.48 ± 2.88	25.51 ± 3.11	0.949
STJ height (mm)	23.29 ± 3.25 (*n* = 232)	23.34 ± 3.24 (*n* = 169)	23.15 ± 3.32 (*n* = 63)	0.694
STJ Perimeter (mm)	91 ± 13.2 (*n* = 231	91.24 ± 9.77 *n* = 168	90.34 ± 10.18 *n* = 63	0.538
LVOT Perimeter (mm)	79.5 ± 9.8 (*n* = 226)	79.8 ± 10.2 (*n* = 164	78.7 ± 8.8 (n = 62)	0.451
RCA height (mm)	17.29 ± 3.16 (*n* = 236)	17.29 ± 3.22	17.30 ± 3.04 (*n* = 64)	0.971
LCA height (mm)	13.4 ± 3.11 (*n* = 234)	13.35 ± 2.90 (*n* = 171)	13.59 ± 3.65 (*n* = 63)	0.573
Calcification parameters				
Visual degree of calcification none Mild Moderate Severe	4 (1.7%) 46 (19.4%) 99 (41.8%) 88 (37.1%)	3 (1.7%) 36 (20.9%) 76 (44.2%) 57 (33.1%)	1 (1.5%) 10 (15.4%) 23 (35.4%) 31 (47.7%)	0.055
Individ. AVC Total (mm^3^)	390.5 (211.1–667.3)	349.5 (179.4–640.4)	461.2 (317.5–790.1)	**0.004**
Individ. AVC LCC (mm^3^)	107.3 (49.6–210.2)	93.95 (46.3–189.5)	146.0 (64.6–248.5)	**0.008**
Individ. AVC RCC (mm^3^)	89.9 (49.35–192.5)	83.35 (43–165.8)	128.2 (63.1–208.4)	**0.027**
Individ. AVC NCC (mm^3^)	154.1 (83.1–298)	139.4 (63.6–263.8)	196.8 (124.1–339.8)	**0.004**
Individ. LVOT calcification (mm^3^)	5.2 (0.1–42) (*n* = 226)	4.35 (0–23.1) (*n* = 164)	16.65(0.3–65.4) (*n* = 62)	**0.024**
Individual threshold (HUs)	600 (550–685)	600 (550–650)	600 (550–700)	0.066

AVC: aortic valve calcification; HUs: Hounsfield units; LCA: left coronary artery; LCC: left coronary cusp; LVOT: left ventricular outflow tract; NCC: noncoronary cusp; RCA: right coronary artery; RCC: right coronary cusp; STJ: sinotubular junction; individ.: individual.

**Table 4:** Hemodynamic measurements overall and in patients with and without post-dilation.

	All Patients (*n* = 237)	Without Post-Dilation (*n* = 172)	With Post-Dilation (*n* = 65)	*p*-Value
SBP pre (mmHg)	141.74 ± 23.5	142.22 ± 23.85	140.48 ± 22.71	0.613
DBP pre (mmHg)	63 (57–72)	63 (58–72)	61 (55.5–70)	0.207
LVEDP pre (mmHg)	21 (16–27)	20.5 (16–26.8)	21 (17–28)	0.643
ARI pre	30.49 ± 9.65	30.69 ± 9.37	28.95 ± 10.4	0.601
SBP post (mmHg)	158.25 ± 24.08	159.59 ± 23.54	154.71 ± 25.28	0.164
DBP post (mmHg)	61 (54–69)	63.5 (57–71.8)	54 (50–59)	**<0.001**
LVEDP post (mmHg)	24 (19–30)	24 (19–29)	24 (19–33)	0.280
ARI post	23.59 ± 8.3	25.26 ± 7.51	19.16 ± 8.73	**<0.001**
ARI ratio post/pre	0.78 (0.61–0.96)	0.82 (0.69–0.99)	0.61 (0.49–0.8)	**<0.001**
ARI ratio ≤ 0.6	55 (23.2%)	23 (13.4%)	32 (49.2%)	**<0.001**
More than mild AR after valve deployment, before PD (angiographically)	61 (25.7%)	3 (1.7%)	58 (89.2%)	**<0.001**

AR: aortic regurgitation; ARI: aortic regurgitation index; DBP: diastolic blood pressure; LVEDP: left ventricular end-diastolic pressure; PD: post-dilation; SBP: systolic blood pressure.

## Data Availability

Data are contained within the article.
